# Exploring medical residents’ perceived need for negotiation skills training

**DOI:** 10.5116/ijme.5c6c.3430

**Published:** 2019-02-28

**Authors:** Lisa N. Isbouts, Arno M.M. Muijtjens, Walther N.K.A. van Mook, Jamiu O. Busari

**Affiliations:** 1 Department of Pediatrics, Radboud University Medical Centre, Nijmegen, the Netherlands; 2 Department of Educational Development and Research, Faculty of Health, Medicine and Life Sciences, Maastricht University, Maastricht, the Netherlands; 3 Academy for Postgraduate Medical Education and Department of Intensive Care Medicine, Maastricht University Medical Center, Maastricht, the Netherlands; 4 Department of Pediatrics, Zuyderland Medical Centre, Heerlen, the Netherlands

**Keywords:** Negotiation skills, management and leadership, competency-based training, medical education, postgraduate

## Abstract

**Objectives:**

This study explores the optimal focus for negotiation skills development training by investigating how often medical residents negotiate in practice, and how they perceive the effectiveness of their negotiation capabilities.

**Methods:**

An exploratory study was performed using a questionnaire regarding the medical residents’ working environment, negotiation frequency, knowledge and skills using a 5-point Likert scale, multiple choice questions and open questions. Exploratory factor analysis with principal component analysis, varimax rotation, reliability analysis, and content analysis were used to reduce the number of variables. Descriptive and interferential statistics and multiple regression analysis were used to analyze the data.

**Results:**

We analyzed the responses of 60 medical residents. The findings showed that the perceived development of their negotiation knowledge (M=3.06, SD=0.83) was less than their negotiation skills (M=3.69, SD=0.47). Their attitude during negotiations, especially females, differed substantially in the interactions with nurses than with their supervisors. Medical residents with more working experience, better negotiation skills or who worked in hierarchical environments negotiated more frequently with their supervisors. Medical residents with better collaboration skills and negotiation knowledge demonstrated better negotiation skills.

**Conclusions:**

This study underlines medical residents’ need for negotiation training. In addition to the basic negotiation knowledge and skills, training programs in negotiation should focus on the medical residents’ awareness of their attitudes during negotiations, combining the assertiveness shown in interactions with supervisors with the empathy and emotional engagement present in interactions with nurses.  Furthermore, attention should be paid to the influence of the environmental hierarchy on negotiation skill development.

## Introduction

In contemporary clinical practice, medical residents and specialists are constantly exposed to changes in health care systems. The complexity associated with these changes places great demands on their management and leadership capabilities.^1^^-^^7^ Unfortunately, most competency-based training programs give little attention to the development of management and leadership skills.^1^^,^^2^^,^^4^^,^^8^^,^^9^ Previous needs assessments in the Netherlands regarding management and leadership development have shown at least half (50%) of the medical residents expressed a need for negotiation training.^1^^,^^2^ These findings have been confirmed by international studies in Canada, Denmark, and Australia.^3^ Teaching medical residents management and leadership skills such as negotiation gives them the ability to cope with ongoing changes in their work.^8^^,^^10^^,^^11^ Despite the need for training in this area, ideal negotiation training programs for medical residents do not yet exist. Moreover, the current literature neither describes how often medical residents negotiate in practice, nor how they perceive the effectiveness of their negotiation capabilities. We believe that information about these issues is needed to design appropriate negotiation training programs. Therefore, it is crucial for program developers to understand the dynamics involved in the process of negotiation amongst medical residents, for example by exploring medical residents’ frequency of negotiation in practice, their perception of their negotiation knowledge and negotiation skills, and their attitude when negotiating with nurses and supervisors. Negotiation knowledge (NK), refers to the extent to which a medical resident is knowledgeable of the various techniques, approaches, and styles of negotiation. Negotiation skills (NS) refer to a resident’s capacity to efficiently carry out a negotiation.

The frequency of medical residents’ negotiations, as well as their NK and NS are expected to vary in relation to the function of the negotiation partner (nurse or supervisor), characteristics of the medical residents themselves, and the medical residents’ working environment. For example, factors that are likely to affect negotiation frequency (NF) are NK and NS.

Medical residents with good NK and NS are likely to negotiate more often than those who lack these capabilities. Collaboration skills (CS), or the ability to build positive working relationships, can also positively influence the cooperation between negotiation partners and their willingness to negotiate.^12^ Older medical residents or medical residents with more working experience (WE) would probably have advantages during negotiations because of their seniority and knowledge about the processes in a hospital. Furthermore, in a working environment with a strong hierarchical character, medical residents are likely to engage less frequently in negotiations.^13^ In such environments, male medical residents are likely to initiate negotiations more frequently and assertively than their female counterparts.^14^

NK also can be positively influenced by NS. Medical residents with proficient CS might easily be able to use NK in different types of situations. Older or more experienced medical residents, for example, are likely to have learned more practice-based NK through practical negotiation experience, which can benefit them during negotiations. Compared to their male counterparts, female medical residents are likely to have different negotiation styles and are more likely to underestimate their knowledge.^14^ Working in an environment with a strong hierarchical character, therefore, could negatively influence NK by providing fewer opportunities to negotiate as well as suppressing the willingness to engage in negotiations.

There are also factors that can optimize NS. For example, adequate NK can positively influence skills such as self-expression and confidence and also stimulate the initiation of the interaction (assertiveness). Good collaborative skills can positively affect NS, as those medical residents with good CS are likely to be better at identifying common goals and brokering agreements than those without it.

Negotiators’ attitudes within a discourse are crucial in determining the outcome of any negotiation. A negotiator could choose to be, among other attitudes, open, empathic, tactful, assertive, emotionally engaged, pragmatic, firm, appeasable, timid, or dominant. Attitude can be influenced by the negotiator’s gender and/or position in a hierarchy. Negotiators of higher status can also have more power in a negotiation.^14^

Since our objective was to design an appropriate training program for negotiation skills development, an exploratory study investigating how often medical residents negotiate in practice, and how they perceive the effectiveness of their negotiation capabilities was designed to address the following research questions:

·       First, how is the frequency of negotiation (NF) with nurses and supervisors related to the medical resident’s NK, NS, CS, WE, age, and gender, and the hierarchical character of the working environment?

·       Second, how is a medical resident’s NK related to NS, CS, WE, age, gender, and hierarchy?

·       Third, how is a medical resident’s NS related to NK, CS, WE, age, gender, and hierarchy?

·       Fourth, how do medical residents perceive their attitude during negotiations (NA)?

## Methods

### Study participants

This study was conducted between September 2015 and January 2016 in the Zuyderland Medical Centre (MC), a 589-bed, large peripheral teaching MC in the Netherlands affiliated with the Maastricht University MC. All medical residents (n=186) enrolled in the institution were eligible for inclusion except those who had a prior negotiation training experience. All medical residents were invited by email to participate in the web-based questionnaire using the online web tool Survey Monkey. The email explained the purpose of the study and we sought and obtained informed consent from the participants through the questionnaire. Participation in the survey was only possible if consent was given.  We emphasized that data gathered during participation would be analyzed anonymously and anyone could opt out of the study at any time for any reason without any consequences. We sought and obtained approval for the study from the Ethical Review Board of the Zuyderland MC.

### Instrument

A 54-item, a web-based questionnaire was designed. The content was validated and improved by two educational experts, superfluous questions eliminated and clarifying remarks added. Two independent medical residents reviewed the questionnaire deleting redundant questions and critically appraising the contents of each question for relevance and comprehension. Following this exercise, the final questionnaire that we used for the survey consisted of 21 items: two open-ended questions, six multiple-choice questions (MCQs) and 13 statements using a five-point Likert scale (see Appendix 1).

The questionnaire items were mapped into three sections. The first section explored the demographic characteristics of the medical resident: age, gender, years of experience as a medical resident, previously received negotiation training (MCQs), department of the medical resident (open question), frequency of negotiations and hierarchy (Likert scale).

The second section assessed the medical resident’s NS (Likert scale) and attitude during negotiations (MCQs). Question regarding NA included the influence of the hierarchical status of negotiation partners (supervisors or nurses) and gender of the medical resident on the medical resident’s attitude to negotiations. To further explore medical residents’ attitude, the respondents were asked to indicate the subset of characteristics that best described their behaviors when negotiating, namely open, tactful, assertive, pragmatic, firm, appeasable, timid, dominant, emphatic, and emotionally engaged.  The third section of the questionnaire focused on the respondents’ self-perceived knowledge of negotiation (Likert scale). The section ended with the contact details of the medical resident (email address, an open question).

**Table 1:** Participants demographics (N=57)

Variables		Mean	SD
1	Male=20, Female=37		
1.1	Age ^a^	2.67	0.83
1.2	Gender (female)	1.65	0.48
1.3	Working experience ^b^	2.80	1.31
1.4	Hierarchy ^c^	2.58	1.07
2	Frequency negotiations: ‘How often do you negotiate…’ ^d^		
	Negotiation frequency with Supervisors	2.14	0.90
2.1	To do an internship?	2.21	0.97
2.2	To go to an international conference?	2.16	1.05
2.3	To go to a course?	2.25	1.07
2.4	To arrange a dedicated time for writing an article/research?	2.00	1.04
	Negotiation frequency with Nurses	3.40	1.06
2.5	When making appointments with the nurses?	3.54	1.04
2.6	To transfer a patient?	3.28	1.20
3	Negotiation knowledge ^c^		
3.1	I know the principles of negotiating.	3.06	0.83
4	Skills: ‘In a negotiation…’		
	Collaboration Skills	3.96	0.63
4.1	I can understand my partner’s position.	3.83	0.65
4.2	Trust in my negotiation partner is important to get to an agreement.	4.10	0.67
	Negotiation Skills	3.69	0.47
4.3	I can express my arguments in a calm and assertive way.	3.73	0.63
4.4	I feel confident.	3.33	0.79
4.5	I can express multiple options.	3.80	0.60
4.6	I can express common goals.	3.90	0.45
4.7	I get to an acceptable agreement for both parties.	3.67	0.55
4.8	I can emphasize the strengths in my arguments.	3.78	0.50
4.9	I can separate the person from the problem.	3.37	0.74

a 38 years (5); b 6 years (5); c Totally disagree (1) – somewhat disagree (2) – not agree/nor disagree (3) – somewhat agree (4) – totally agree (5) ; d (Almost) never (1) – rarely (2) – sometimes (3) – regularly (4) – (almost) always (5)

### Scale construction

As a large number of variables were included in our questionnaire, an exploratory factor analysis (EFA) to identify any underlying relationships between the different questions was performed, a technique which helps to identify subsets of related items often referred to as scales of the questionnaire. Our goal was to identify, if present, any latent constructs underlying these scales or clusters of measured variables. As we did not have a separate a priori hypothesis of the patterns of the variables we measured, performing an EFA would help determine which variables we eventually could use in our analysis.  Clusters of items related to the same content were sought for using principal component analysis, varimax rotation, reliability analysis, and content analysis. Separate EFAs were executed for the sets of items regarding skills (Table 1, 4.1-4.9), and NF (Table 1, 2.1-2.6). To test the reliability and remove items that did not contribute to the result, a reliability analysis was performed for each scale. A Cronbach’s alpha of >0.60 was considered to indicate a sufficient level of reliability.

**Table 2:** Factor loadings and resulting scales of the Explorative Factor Analysis for the negotiation skills items

Items	Factor 1	Factor 2
Collaboration skills		
I can understand my partner’s position.	**0.83**	0.23
Trust in my negotiation partner is important to get to an agreement.	**0.79**	0.014
Negotiation skills		
I can express goals in common.	0.55	**0.67**
I can express my arguments in a calm and assertive way.	0.19	**0.77**
I feel confident.	-0.40	**0.79**
I can express multiple options.	0.35	**0.76**

### Data analysis

#### Descriptive and interferential statistics

Descriptive and interferential statistics were applied to describe the age, gender, working experience of the participants as well as the level of hierarchy in the departments.

#### Exploratory factor analysis

EFA regarding NS: A 3-factor solution was initially found based on the criterion eigenvalue >1.0. Items 4.7-4.9 were removed because of communalities <0.5. The EFA was repeated, and a satisfactory 2-factor solution was found based on eigenvalue >1.0, the scree-plot, and the content of the items. Table 2 shows the factor loadings of the items on the two scales, which are marked as “collaboration skills” and “negotiation skills”. Table 3 shows the Cronbach’s Alpha and the value for the corresponding variable defined as the mean of the scores of the scale’s items.

EFA regarding NF: The initial EFA resulted in a 2-factor solution. Item 2.1 was removed because of low communality, and the final EFA resulted in a 2-factor solution based on eigenvalue >1.0, the scree-plot, and the content of the items in relation to the hierarchical position of the negotiation partner. Table 4 shows the factor loadings of the items on the two scales, which are marked as “negotiation frequency with supervisors” and “negotiation frequency with nurses”. Table 3 shows the Cronbach’s Alpha and the value for the corresponding variable defined as the mean of the scores of the scale’s items.

**Table 3:** Descriptive statistics for collaboration and negotiation skills

Variables	No. Items	Cronbach’s alpha	M (SD)	% of variance^a^
Collaboration skills	2	0.62	7.92 (1.25)	32.00
Negotiation skills	4	0.74	14.77 (1.89)	38.31
Negotiation frequency with Supervisors	3	0.82	6.43 (2.71)	44.07
Negotiation frequency with Nurses	2	0.84	6.80 (2.11)	37.71

^a^After varimax rotation.

##### Multiple regression analysis

To answer the first research question i.e. ‘How is the frequency of negotiation (NF) with nurses and supervisors related to the medical resident’s NK, NS, CS, WE, age, and gender, and the hierarchical character of the working environment?’, two multiple regression analysis (MR analysis) were performed, one for negotiation frequency with supervisors and one for negotiations with nurses. Independent variables were NK, CS, NS, age, gender, and hierarchy.

To answer the second i.e. ‘How is a medical resident’s NK related to NS, CS, WE, age, gender, and hierarchy?’ and third research questions, i.e., ‘How is a medical resident’s NS related to NK, CS, WE, age, gender, and hierarchy?’ two MR analysis were performed with the set of independent variables indicated above.

**Table 4:** Factor loadings and resulting scales of the Explorative Factor Analysis for the negotiation frequency items

Items	Factor 1	Factor 2
Negotiation frequency with Supervisors		
To go to an international conference?	**0.89**	0.10
To go to a course?	**0.90**	0.11
To arrange a dedicated time for writing an article/research?	**0.76**	0.12
Negotiation frequency with Nurses		
When making appointments with the nurses?	0.12	**0.92**
To transfer a patient?	0.13	**0.92**

The MR analysis was performed using a backward procedure, stepwise excluding independent variables that did not contribute sufficiently to the explained variance of the dependent variable. A p-value of <0.05 was considered to indicate a significant effect. The standardized regression coefficient (beta) resulting from the MR analysis was used as an indicator of effect size classified according to Cohen’s values (0.1, 0.3 and 0.5, respectively corresponding to a small, moderate and large effect).^15^ The self-perceived attitude of medical residents during negotiations (variable NA, research question 4) was analyzed by calculating the percentage of medical residents who indicated a characteristic to be representative of their attitude. These percentages were calculated separately for negotiations with nurses, and supervisors, and male and female medical residents. The Statistical Package for Social Sciences (SPSS) for Windows was used for scale construction and data analysis.

**Table 5:** Results of the multiple regression analysis

Independent variables^d^	Dependent variables								
	Negotiation frequency with supervisors			Negotiation knowledge			Negotiation skills		
	b^a^	beta^b^	p^c^	b	beta	p	b	beta	p
Working experience	0.21	0.31	0.018						
Hierarchy	0.27	0.32	0.020				-0.09	-0.21	0.050
Negotiation skills	0.62	0.33	0.017	1.1	0.63	0.001			
Negotiation knowledge							0.34	0.60	0.001
Collaboration skills							0.23	0.27	0.014

^a^b: regression coefficient; ^b^beta: standard regression coefficient; ^c^p: two-sided p-value of the t-test for b; ^d^Only significant effects are shown; Gender and Age were included in the analysis but did not show any significant effect.

## Results

### Demographics

Out of 186 medical residents about one third responded (60 medical residents). Three medical residents (5%) were excluded because of prior training in negotiation. Most respondents were between 24-33 years old and female (65%, (37/57)).^16^ The majority of the medical residents had around 2.5 years of WE (Table 1). In most of the training programs in the hospital (80%, 12/15), male supervisors among clinical supervisors (teaching staff) predominated (79%) while in three programs (gynecology, pediatrics, and emergency medicine) a female predominance of the teaching staff was observed (75%). In terms of departmental hierarchy, the presence of a strong hierarchical working environment was rated with a mean of 2.58 (SD=1.07) on a 5-point Likert scale, indicating that the working environment was, on average, mildly hierarchical.

### Negotiation frequency

For the statements pertaining to negotiation frequency with supervisors and nurses (Table 1), the mean scores were 2.13 (SD=0.90) and 3.42 (SD=1.04) respectively, indicating that medical residents negotiate more often with nurses than with supervisors.

Multiple regression analysis for negotiation frequency with supervisors showed that medical residents with more WE, in a more hierarchical working environment, or with better NS, negotiated with supervisors more frequently (b=0.21, 0.27, and 0.62 respectively), with standard regression coefficients (beta) ranging from 0.31 to 0.33 indicating moderate effect sizes (Table 5).

None of the independent variables in the multiple regression analysis for negotiation frequency with nurses showed a significant effect. Therefore, the results of this analysis were not included in Table 5.

### Negotiation knowledge and skills

Medical residents perceived their NK as moderate (Table 1, M=3.06, SD=0.83). They have a more positive perception regarding their CS (M=3.96, SD=0.56) and NS (M=3.69, SD=0.47). Multiple regression analysis of negotiation knowledge showed that medical residents with better NS were found to score significantly higher on the scale of NK (b=1.10) (Table 5). Multiple regression analysis of negotiation skills resulted in medical residents with better NK or better CS, scoring significantly higher on the scale of NS (Table 5, respectively b=0.34 and 0.23). A more hierarchical working environment had an almost-significant negative impact on NS with a small effect size (beta) of -0.21 (b= -0.09) and a p-value of 0.05.

### Negotiation attitude

Male and female medical residents reported very different attitudes during negotiations with nurses compared to negotiation with supervisors.

### Nurses versus supervisors

Medical residents identified their attitudes toward nurses mostly as open, empathic, and tactful. Moreover, a relatively large proportion (39% (22/57)) identified themselves as emotionally engaged in these negotiations. Towards supervisors, medical residents identified themselves as assertive, open, and tactful. However, only a quarter (26%, 15/57) of the medical residents identified themselves as empathic when negotiating with supervisors, compared to more than half (67%, 38/57) when negotiating with nurses. Furthermore, only a few (18%, 10/57) medical residents identified themselves as emotionally engaged in negotiations with supervisors, less than half compared to negotiations with nurses. When negotiating with supervisors, medical residents indicated that they were more assertive and appeasable, but also more timid.

### Gender

Regarding NA, large differences were observed between male and female medical residents. Female medical residents considered themselves more open, empathetic, assertive, emotionally engaged, and firm toward nurses than male medical residents did. However, both male and female medical residents reported themselves to be more timid, less emotionally engaged, and less empathic towards their supervisors. Female medical residents showed a significant decrease compared to males in having an empathic attitude, from nearly half (47%, 27/57) towards nurses to very few (14%, 8/57) towards supervisors. Males reported themselves to be twice as assertive toward supervisors than nurses, whereas females were about equally assertive towards both. Females rated themselves as more timid and appeasable towards supervisors, while males were more appeasable to nurses.

## Discussion

The objectives of the current exploratory study were to analyze the medical residents’ perception of when they negotiate and their level of proficiency in effective negotiation relating to knowledge, skills, and attitude. Our results indicate that very few (5%, 3/60) of all medical residents had ever attended negotiation training, which is in line with the need for negotiation training in the existing literature.^1^^,^^2^^, ^^17^^-^^19^ In the following sections, we discuss the main findings and implications regarding the medical residents’ NF, NK, NS, and NA during negotiations.

With respect to when medical residents negotiate (negotiation frequency), it became apparent that medical residents interacted more with nurses than with their supervisors. Nurses expect female medical residents to be more sympathetic and communicative as described by Linden et al.^20^ This could explain why medical residents interact more often with nurses than with supervisors to build trust and a good relationship.^20^^-^^22^ Even though medical residents interacted more frequently with nurses, negotiation frequency did not vary with WE, hierarchy, NS, NK or CS. This may be because nurses and medical residents do not have a choice whether or not to have these negotiations.

The frequency of medical residents’ interaction with supervisors was subject to their NS. Medical residents with better skills negotiated more often with supervisors, probably because of their higher level of confidence, which could also explain why more experienced medical residents negotiated more often. This is consistent with the findings of Olmos-Vega and colleagues who described how medical residents use negotiation as an adaptation strategy to their day-to-day changing supervisors and how they as their skills increase form their own working repertoire.^23^ This observation advocates for the incorporation of negotiation training in the first years of residency to improve medical residents’ NS, which we anticipate would have a positive influence on the frequency of negotiations, interaction with supervisors^23^ and empower medical residents in their future careers. Surprisingly, a hierarchical working environment was found to stimulate medical residents to negotiate more often with their supervisors. This is in contrast to the findings of Olde Bekkink and colleagues who described hierarchy as a continuous barrier in interprofessional communication.^13^ However, we believe that hierarchical environments increase medical residents’ awareness of the strength of their negotiation position, which in turn enables them to speak up for themselves and draw attention to their abilities as future specialists.

The majority of the medical residents had moderate knowledge of the principles of negotiation, i.e. (negotiation knowledge). We found that perceived levels of NK and NS were strongly correlated (beta=0.60), indicating that training medical residents in NK and in separate NS will each positively influence the other.

Besides the positive influence of NK, negotiation skills are also enhanced by collaborative skills. Medical residents with good collaborative skills are better at identifying common goals and remaining calm because they have a better understanding of their negotiation partner. Interestingly, it seems that a predominant hierarchical structure in the medical residents working environment had a small negative effect on the medical residents’ perception of their NS. Possibly medical residents feel more dependent on their negotiation partner in a hierarchical working environment that leads them to hold back during negotiations and therefore appreciate their NS less. Another possible explanation is that medical residents negotiate more in a hierarchical working environment but under the social pressures of the hierarchy the quality of their interactions as well as the quality of their NS-decreases, which is in line with the findings of Olde Bekkink et al.^13^

Despite the fact that more experienced medical residents negotiate more often, they did not score significantly higher on NS. This could be because they know their own limitations and place higher demands on themselves during negotiations, therefore scoring themselves lower on the survey.

The results also show differences in attitudes toward the position of the negotiation partner and more gender-specific attitudes, i.e. (negotiation attitude).

First, there are important differences in medical residents’ attitudes towards nurses and supervisors. Medical residents are more emphatic and emotionally engaged toward nurses and more assertive, timid, and appeasable toward their supervisors. This is consistent with the result that a hierarchic working environment stimulates NF but impairs the quality of medical residents’ NS.  Second, male and female medical residents have different attitudes during negotiations. This is consistent with the findings of Linden et al who describe that female medical residents prefer a more communicative and collaborative style.^20^ During interactions with supervisors, medical residents - especially female medical residents - were alarmingly less empathetic and emotionally engaged than in their interactions with nurses. With more than half (65%, 37/57) female medical residents, it is possible that this difference is linked to the majority male supervisors in the hospital, which would be consistent with the findings of Linden et al.^20^ The male to female supervisors ratio will undoubtedly change further as female medical residents finish their training and become supervisors themselves. This could potentially alter the perceived departmental hierarchy,^24^ NA, and level of NS.

### Limitations

This study has several limitations. There was no existing theoretical framework or previous studies on which we could rely on making our questionnaire. The perspective on negotiation training was only investigated on the basis of the perception of the medical residents. Hence, objective data were not available. Due to the different nature of their jobs, the content of the questions regarding nurses and supervisors differed. Questions regarding supervisors were mainly about time management and preferred training, while questions regarding nurses were about direct patient care. Thus, negotiations with nurses and supervisors might necessitate different kinds of negotiation skills. This has to be taken into account when considering the difference in answers between these two groups of questions.

Concerning the reliability of the scales, in general values of Cronbach’s Alpha between 0.70-0.95 are accepted.^25^ We accepted a Cronbach’s Alpha of >0.60 because our sample size was relatively small for an EFA and because we were unable to test the validity of our questionnaire due to the lack of previous research regarding this subject. Despite these limitations, we considered reliability and internal consistency to be important aspects of validity.

Finally, the limited response rate may have introduced potential selection bias, with medical residents less interested in management and leadership development or feeling more time-pressured not responding to the questionnaire. Nevertheless, the male-to-female ratio was representative for the Netherlands.

## Conclusions

This study shows that only 5% of medical residents ever attended negotiation training. The medical residents perceived their Negotiation Knowledge (NK) and Negotiation Skills (NS) to be moderate. In addition to the need for basic knowledge and skills regarding negotiation, a need for training programs focusing on the dynamics between gender, hierarchies, and attitude during negotiations was voiced. Our findings suggest that NK and NS have positive reciprocal and synergistic effects on each other, and the presence of NS in medical residents enhances their NF.

Two important steps in medical education are needed to improve medical residents’ negotiation capabilities. Firstly, teaching them to be aware of their attitudes during negotiation. Secondly, combining the assertiveness shown in interactions with supervisors with the empathy and emotional engagement present in interactions with nurses. Improving knowledge, skills, and attitude regarding negotiation will potentially help healthcare professionals tackle changes in the health care system more efficient. Future studies should focus on the development of a suitable training method to address the perceived needs.

### Conflict of Interest

The authors declare that they have no conflict of interest.

## Supplementary material

Supplementary file 1 Appendix 1. Web-based questionnaire: Medical residents’ perceived need for negotiation training in postgraduate medical training
