# Supplementary material for: Exploring medical residents’ perceived need for negotiation skills training
Source: Int J Med Educ. 2019 Feb 28;10:45–53. doi: 10.5116/ijme.5c6c.3430 (PMC6766400; doi:10.5116/ijme.5c6c.3430)
Supplement: Supplementary file 1 — Appendix 1. Web-based questionnaire: Medical residents’ perceived need for negotiation training in postgraduate medical training [file ijme-10-45-S1.pdf]

Appendix 1

Web-based questionnaire:
Medical residents' perceived need for negotiation training in postgraduate medical training

A. Demographics

Age

- <24 years (1)
- 24-28 years (2)
- 29-33 years (3)
- 34-38 years (4)
- >38 years (5)

Gender

- Male (1)
- Female (2)

Years of experience as a medical resident

- <1 year (1)
- 1-2 years (2)
- 3-4 years (3)
- 5-6 years (4)
- >6 years (5)

Previous negotiation training

- Yes (1)
- No (2)

Department (open question)

How often do you negotiate?

- To go to an international conference?
- To go to a course?
- To do an internship?
- To arrange a dedicated time for writing an article/research?
- (Almost) never (1)
- Rarely (2)
- Sometimes (3)
- Regularly (4)
- (Almost) always (5)

There is a strong hierarchy in my working environment:

- Totally disagree (1)
- Somewhat disagree (2)
- Not agree/nor disagree (3)
- Somewhat agree (4)
- Totally agree (5)

B. Skills and attitude

Skill statements: In a negotiation…

- I can understand my partner's position.
- I can express common goals.
- Trust in my negotiation partner is important to get to an agreement.
- I can express multiple options.
- I can express my argumentation in a calm and assertive way.
- I feel confident.
- I get to an acceptable agreement for both parties.

Possible answers on a 5-point Likert scale:

- Totally disagree (1)
- Somewhat disagree (2)
- Not agree/nor disagree (3)

Somewhat agree (4)<br>Totally agree (5)
Negotiation attitude towards nurses:
Open<br>Empathic<br>Tactful<br>Assertive<br>Emotionally engaged<br>Pragmatic<br>Firm<br>Appeasable<br>Timid<br>Dominant
Select the attitudes that you present in negotiation with nurses (multiple choice question)
Negotiation attitude towards supervisors:<br>Open<br>Empathic<br>Tactful<br>Assertive<br>Emotionally engaged<br>Pragmatic<br>Firm<br>Appeaseable<br>Timid<br>Dominant

**C.** Knowledge
I have knowledge about the principles of negotiation (possible answers on a 5-point Likert scale)
Totally disagree (1)<br>Somewhat disagree (2)<br>Not agree/nor disagree (3)<br>Somewhat agree (4)<br>Totally agree (5)
Email address (open question)
